# Perceptual and conceptual processing of visual objects across the adult lifespan

**DOI:** 10.1038/s41598-019-50254-5

**Published:** 2019-09-24

**Authors:** Rose Bruffaerts, Lorraine K. Tyler, Meredith Shafto, Kamen A. Tsvetanov, Cambridge Centre for Ageing and Neuroscience, Carol Brayne, Edward T. Bullmore, Andrew C. Calder, Rhodri Cusack, Tim Dalgleish, John Duncan, Richard N. Henson, Fiona E. Matthews, William D. Marslen-Wilson, James B. Rowe, Karen Campbell, Teresa Cheung, Simon Davis, Linda Geerligs, Rogier Kievit, Anna McCarrey, Abdur Mustafa, Darren Price, David Samu, Jason R. Taylor, Matthias Treder, Janna van Belle, Nitin Williams, Lauren Bates, Tina Emery, Sharon Erzinçlioglu, Andrew Gadie, Sofia Gerbase, Stanimira Georgieva, Claire Hanley, Beth Parkin, David Troy, Tibor Auer, Marta Correia, Lu Gao, Emma Green, Rafael Henriques, Jodie Allen, Gillian Amery, Liana Amunts, Anne Barcroft, Amanda Castle, Cheryl Dias, Jonathan Dowrick, Melissa Fair, Hayley Fisher, Anna Goulding, Adarsh Grewal, Geoff Hale, Andrew Hilton, Frances Johnson, Patricia Johnston, Thea Kavanagh-Williamson, Magdalena Kwasniewska, Alison McMinn, Kim Norman, Jessica Penrose, Fiona Roby, Diane Rowland, John Sargeant, Maggie Squire, Beth Stevens, Aldabra Stoddart, Cheryl Stone, Tracy Thompson, Ozlem Yazlik, Dan Barnes, Marie Dixon, Jaya Hillman, Joanne Mitchell, Laura Villis, Alex Clarke

**Affiliations:** 1 Department of Psychology, University of Cambridge, Cambridge, CB2 3EB UK; 2 Laboratory for Cognitive Neurology, Department of Neurosciences, University of Leuven, 3000 Leuven, Belgium; 3 Neurology Department, University Hospitals Leuven, 3000 Leuven, Belgium; 4 Cambridge Centre for Ageing and Neuroscience (Cam-CAN), University of Cambridge and MRC Cognition and Brain Sciences Unit, Cambridge, CB2 7EF UK

**Keywords:** Language, Perception

## Abstract

Making sense of the external world is vital for multiple domains of cognition, and so it is crucial that object recognition is maintained across the lifespan. We investigated age differences in perceptual and conceptual processing of visual objects in a population-derived sample of 85 healthy adults (24–87 years old) by relating measures of object processing to cognition across the lifespan. Magnetoencephalography (MEG) was recorded during a picture naming task to provide a direct measure of neural activity, that is not confounded by age-related vascular changes. Multiple linear regression was used to estimate neural responsivity for each individual, namely the capacity to represent visual or semantic information relating to the pictures. We find that the capacity to represent semantic information is linked to higher naming accuracy, a measure of task-specific performance. In mature adults, the capacity to represent semantic information also correlated with higher levels of fluid intelligence, reflecting domain-general performance. In contrast, the latency of visual processing did not relate to measures of cognition. These results indicate that neural responsivity measures relate to naming accuracy and fluid intelligence. We propose that maintaining neural responsivity in older age confers benefits in task-related and domain-general cognitive processes, supporting the brain maintenance view of healthy cognitive ageing.

## Introduction

Recognizing objects is a fundamental aspect of human cognition. Accessing the meaning of an object is essential in order to interact successfully with the world around us, and is therefore a vitally important cognitive function to maintain across the adult lifespan. Research with young adults suggests that accessing meaning from vision is accomplished within the first half second of seeing an object^1–9^, and involves recurrent activity within the ventral temporal cortex extending into the anteromedial temporal cortex^2,10–14^. As the visual input is processed along this pathway, it is transformed into an initial coarse grained semantic representation (e.g. *animal, tool*) in the inferior temporal cortex before a more semantically specific representation emerges (e.g. *cow, hammer*) in the anteromedial temporal cortex^12,15,16^.

Using multivariate analysis enables quantification of the representation of perceptual and semantic information during this rapid transformation process. Clarke *et al*.^4^ investigated the time course of single object processing using a computational model of vision^17^ combined with semantic-feature information^18^. In young participants, perceptual information was represented within the first 150 ms of object presentation, with the addition of semantic information providing a better account of object representations up to 400 ms^4^. The combination of explicit models of vision and semantics provides an integrated account of the processing of perceptual and conceptual information of visual objects^19,20^. While it is well-known that visual processing becomes slower in middle-aged and mature people^21–23^, it is unclear whether there are age-related differences in the processing of visual or semantic information of single objects. Here, we evaluated differences in measures of perceptual and semantic information across the lifespan using MEG in a large population-derived ageing cohort from the Cambridge Centre for Ageing and Neuroscience (Cam-CAN; http://www.cam-can.org). Possible age-related neural differences in object processing may or may not relate to behavior: changes may impact either task-performance or domain-general cognitive function, or both. To address this, we relate neural measures of perceptual and semantic information processing to different metrics of cognition to evaluate their relevance for healthy cognitive ageing.

It is well established that both early and late aspects of visually evoked neural responses show age-related changes, where activity is reduced and delayed with age^22,24–28^. For example, recently Price *et al*.^28^ used MEG to show that the initial neural response to checkerboards in early visual cortex is increasingly delayed across the lifespan. Further, age-related differences in later visual components have been observed, such as delayed N170^25^ and slower information processing of faces^26,27^. However, it remains to be determined if age-related changes are related to early visual processes or semantic activation, how changes in the initial visual processes (amplitude or delay) impact semantics, and further if such changes have behavioural consequences.

Rather than a mere description of age-related neural differences, a challenge is to relate these differences to cognition to elucidate what happens during successful ageing^29,30^. Across the adult lifespan, differences in fluid intelligence and picture naming accuracy can be predicted from the degree to which different brain networks are responsive to these tasks^31^. This suggests that maintenance of neural responsivity could support successful cognitive ageing. The “maintenance view” hypothesizes that the brains of mature adults whose neurobiology is well preserved, will show activation patterns similar to younger adults which are germane to proficient performance^32^. However, many current models of healthy cognitive ageing are primarily based on fMRI studies^29^, which could be confounded by the effects of age on vasculature^33^. Therefore, we need electrophysiological studies to complement fMRI research, and extend our current theoretical models of neurocognitive ageing. Research techniques such as MEG, provide both a direct measure of neural activity and allow us to examine temporal dynamics, and therefore offer an ideal approach to examine neurocognitive models of ageing.

In the current study, we ask whether the representation of perceptual and semantic information reflected in the MEG signal is different across the adult lifespan, and whether this relates to task–related measures of cognition, e.g. naming accuracy, and domain-general cognitive measures, namely fluid and crystallized intelligence. We analyzed MEG signals during a picture naming task from the Cam-CAN cohort study^34^. By relating single-object measures of vision and semantics to MEG signals, we were able to test (1) whether representations of visual and semantic information are different across the adult lifespan (2) whether changes in representation of visual information impacts semantics, and (3) do these age-related differences in neural processing relate to behavioral performance.

Rather than using an approach based on raw MEG signals, we follow the strategy used in our previous study^4^ where we modelled MEG signals with explicit models of vision and semantics. The outcome, which is a quantification of the individual’s capacity to represent visual or semantic information, can be seen as a measure of neural responsivity. In other words, the individual’s ability to neurally represent a stimulus – quantified by a higher correlation between neural activity and the visual or semantic model - implies higher neural responsivity. The brain maintenance hypothesis suggests that better neural responsivity supports better cognition in older individuals^32^, and we predict that we will see evidence of this through our measures of visual and semantic processing. Moreover, moderation analysis can be used to test whether age plays a role in the relationship between neural responsivity and behavioral performance. Following Samu *et al*.^31^, we investigated picture naming accuracy, a task-specific cognitive measure of object naming, which is based on the output of visual and semantic information processing. Additionally, we investigated domain-general performance (fluid and crystallized intelligence) because neural responsivity might reflect a more general neural property of performance across tasks. Fluid intelligence is on average lower in mature adults, while crystallized intelligence is unchanged^31,35,36^. This difference prompts us to study the relationship between neural responsivity and both cognitive measures.

## Results

### Behavioural results

**Figure 1:**
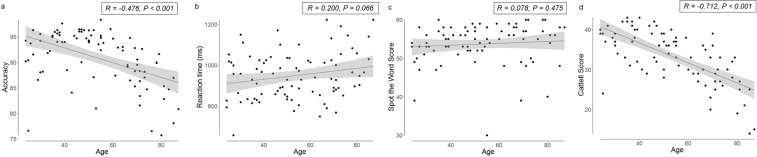
Behavioural results for (**a**) naming accuracy, (**b**) reaction times, (**c**) Spot the Word and (**d**) Cattell Culture Fair versus age.

Overall object naming accuracy for the 302 common objects was high (90.9%, SD 5.3%), but decreased significantly with age (Pearson’s r = −0.476, p < 0.001) (Fig. 1a). When dividing the participants into equally sized age groups, we found mean accuracy was 92.3% (SD 4.7%) in the young group (24–37 years old), in the middle-aged group (47–60 years old) it was 93.8% (SD 3.8%) and in the mature group (70–87 years old) it was 85.4% (SD 4.8%). These results are consistent with previously reported age-related differences in accuracy for the same participants during fMRI picture naming^31^. Mean reaction times for correct responses tended to increase with age, but did not reach significance (r = 0.200, p = 0.066; Fig. 1b).

Crystallized intelligence (measured with the Spot the Word task^37^) did not change with age in our sample (r = 0.078, p = 0.475, Fig. 1c). As expected, fluid intelligence (measured with Cattell Culture Fair^38^) significantly declined with age (r = −0.712, p < 0.001, Fig. 1d). Crystallized intelligence and fluid intelligence correlated with naming accuracy (resp. r = 0.246, p = 0.023; r = 0.560, p < 0.001). Mean reaction times for correct responses were faster when fluid intelligence scores were higher (r = −0.281, p = 0.009). Mean reaction times for correct responses did not correlate to crystallized intelligence (r = −0.143, p = 0.189).

### Visual and semantic model fits decrease across the lifespan

We next evaluated differences in visual and semantic neural processes across the lifespan by quantifying how much of the variability in the MEG signals could be explained by the models of vision and semantics – namely the AlexNet Deep Convolutional Neural Network^39^ and a semantic feature-based model^18,40^. Regularised regression was performed at each time-point and for every MEG sensor separately, providing a measure of how well the visual or semantic models could explain the MEG signals over time.

**Figure 2:**
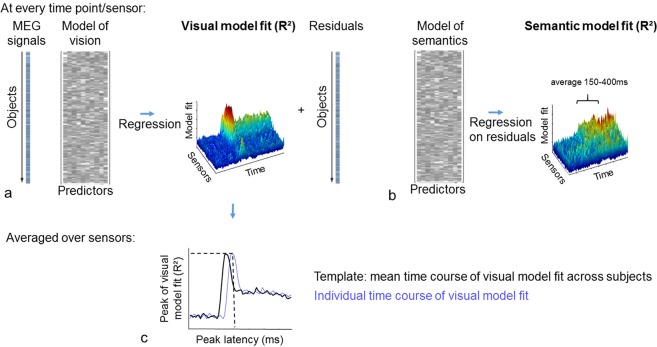
Schematic representation of the analysis pipeline. Calculation of (**a**) visual model fit, (**b**) semantic model fit and (**c**) peak latency. See method section for details.

First, using the fit between the visual model and the MEG signals, we calculated a single measure of the individual’s visual model fit (Fig. 2a), and an individual peak latency (Fig. 2c). After removing effects attributed to the visual model (Fig. 2a), a second regression was used to calculate how well the semantic-feature based model could explain the residual MEG signals over time (after accounting for the visual model, Fig. 2b). The individual semantic model fit was determined as the average semantic model fit between 150 and 400 ms (interval derived in an independent sample^4^). Using this approach, we obtained independent measures of visual and semantic model fits for each individual.

**Figure 3:**
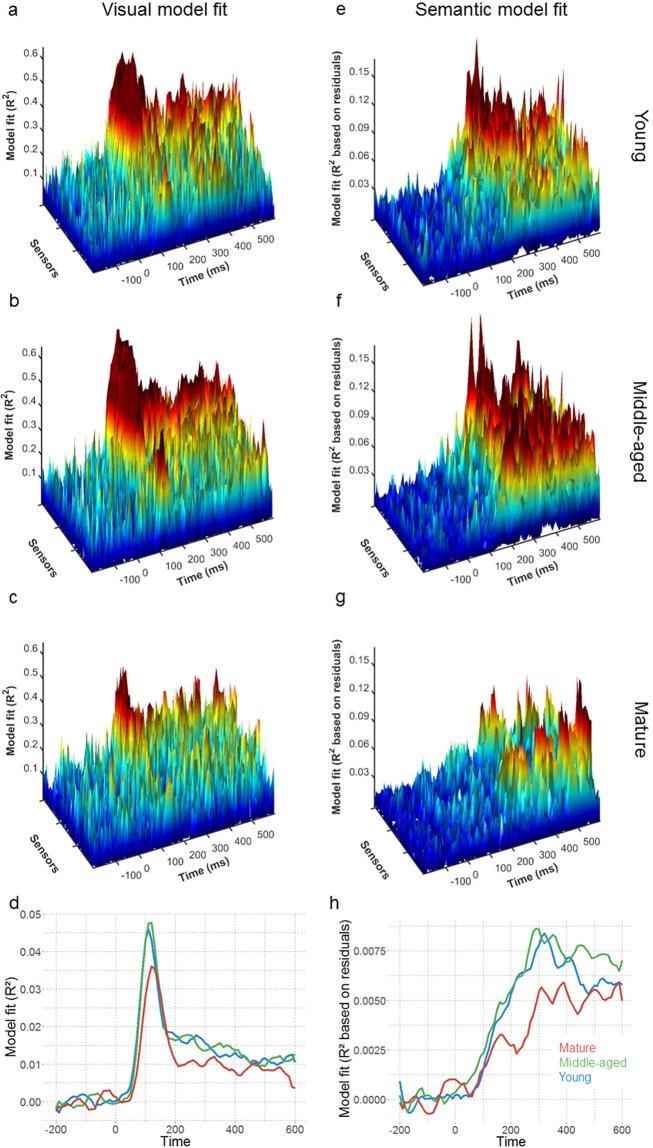
Model fits across time showing R² values for the (abcd) visual and (efgh) semantic model for the (ae) young, (bf) middle-aged and (cg) mature groups for all sensors and averaged across sensors for the three age groups (dh). Note that the effect sizes cannot be directly compared, as the visual model fit is calculated on the raw MEG signal and the semantic model fit is calculated on the residuals after the visual model fits are regressed out (see methods and Fig. 2).

**Figure 4:**
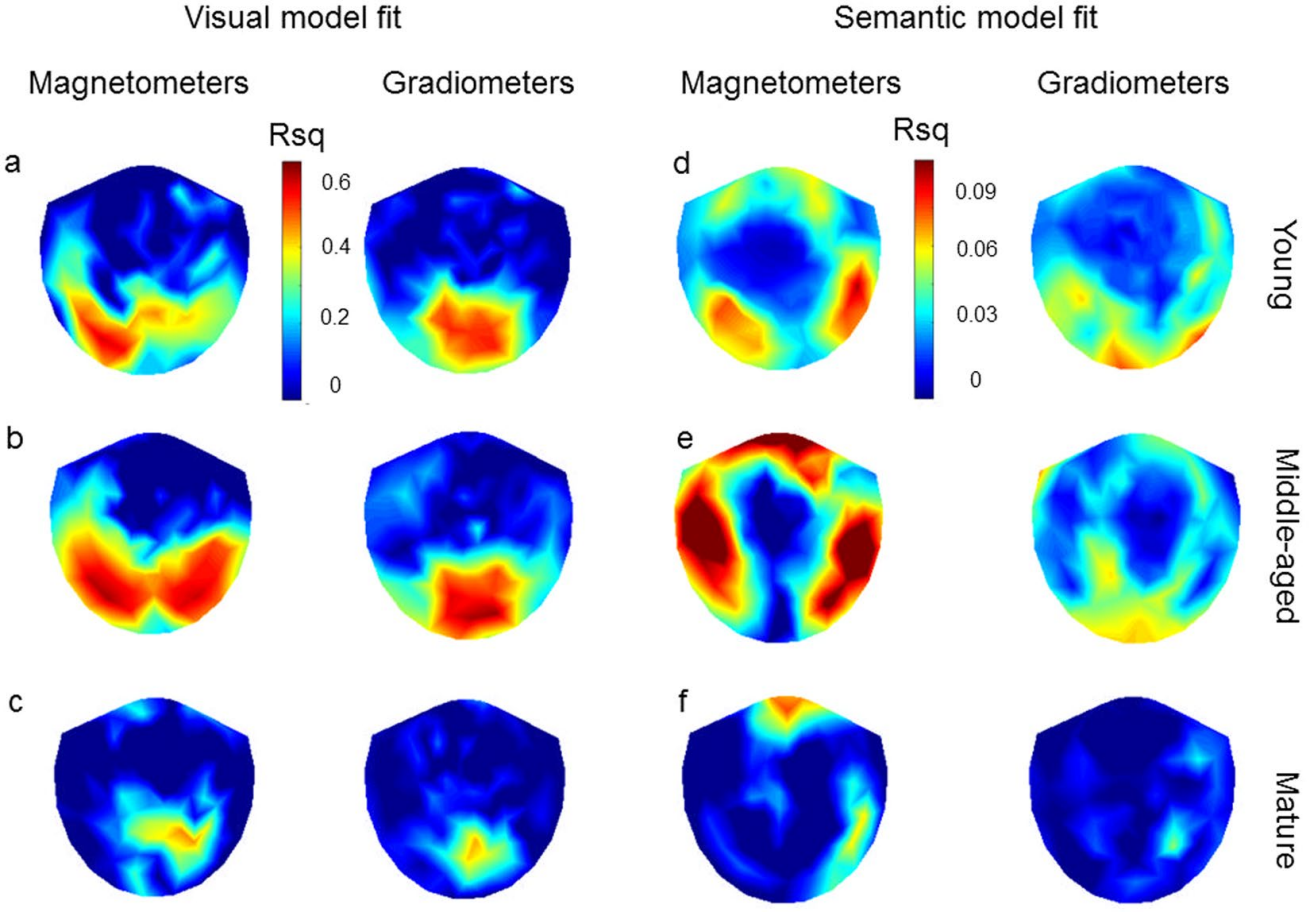
Topographies of visual model fit at 110 ms after stimulus onset, the mean peak latency, (abc) and semantic model fit at 290 ms after stimulus onset, derived from Clarke *et al*. (2015) as time with maximal classification accuracy for the semantic model, (def). Topographies for magnetometers gradiometers are visualized in the young (ad), middle-aged (be) and mature (cf) age groups.

**Figure 5:**
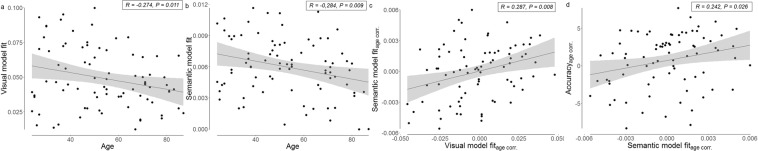
Relationship between the visual model fit, the semantic model fit, age and accuracy. (**a**) Correlation between age and the visual model fit, (**b**) Correlation between age and the semantic model fit, (**c**) Correlation between visual and semantic model fit (corrected for age), (**d**) Correlation between accuracy and semantic model fit (corrected for age).

Overall, we see positive visual model fits across all ages peaking close to 110 ms (Fig. 3a–d), with the greatest model fits over posterior sensors (Fig. 4a–c). The visual model fit significantly decreased across the adult lifespan (r = −0.274, p = 0.011; Figs 3d and 5a) indicating that the capacity to represent visual information, as reflected by the AlexNet model, is reduced in the mature group. Across all age groups, the semantic model demonstrated increasing model fits between 150 and 400 ms (Fig. 3e–h), with the highest model fits observed over temporal sensors (Fig. 4d–f). Semantic model fits significantly decreased with age (r = −0.284, p = 0.009; Figs 3h and 5b). Variability for the semantic model fits do not change across the lifespan (Fligner-Killeen test of homogeneity of variances: p = 0.593), whilst for the visual model fit, variability was lower in the mature group (p = 0.002).

A key question is whether the visual model fit influences the semantic model fit, and how model fits relate to task performance. We found a significant positive correlation between the visual and semantic model fits (r = 0.353, p < 0.001), which remained even after controlling for age (r = 0.287, p = 0.008) (Fig. 5c). This shows that the initial visual representation of an item has subsequent consequences for its semantic representation, over and above the age-related differences. Further, we observed that higher semantic model fits correlated with higher naming accuracy levels, over and above the effect of age (r = 0.242, p = 0.026; Fig. 5d). This effect was not present for the visual models fits (r = 0.122, p = 0.264, not shown). No correlation was found between visual or semantic model fits and domain-general performance measures namely Cattell Score and Spot the Word score (p > 0.247).

### Effect of age on the relationship between performance and visual and semantic model fits

Having a higher semantic model fit related to better accuracy for object naming. Next we ask whether the relationship between either of our measures of neural responsivity, the visual and semantic model fits, and cognition is different across the age groups using moderation analysis. Moderation analysis determines whether the relationship between the independent variable (e.g. visual model fit) and a dependent variable (e.g. accuracy) varies as a function of another dependent variable, i.e. moderator variable (e.g. age). In terms of the brain maintenance view, it would be expected that when the visual and semantic model fits are higher, and therefore more like the younger and middle-aged participants, cognitive performance should be better.

**Figure 6:**
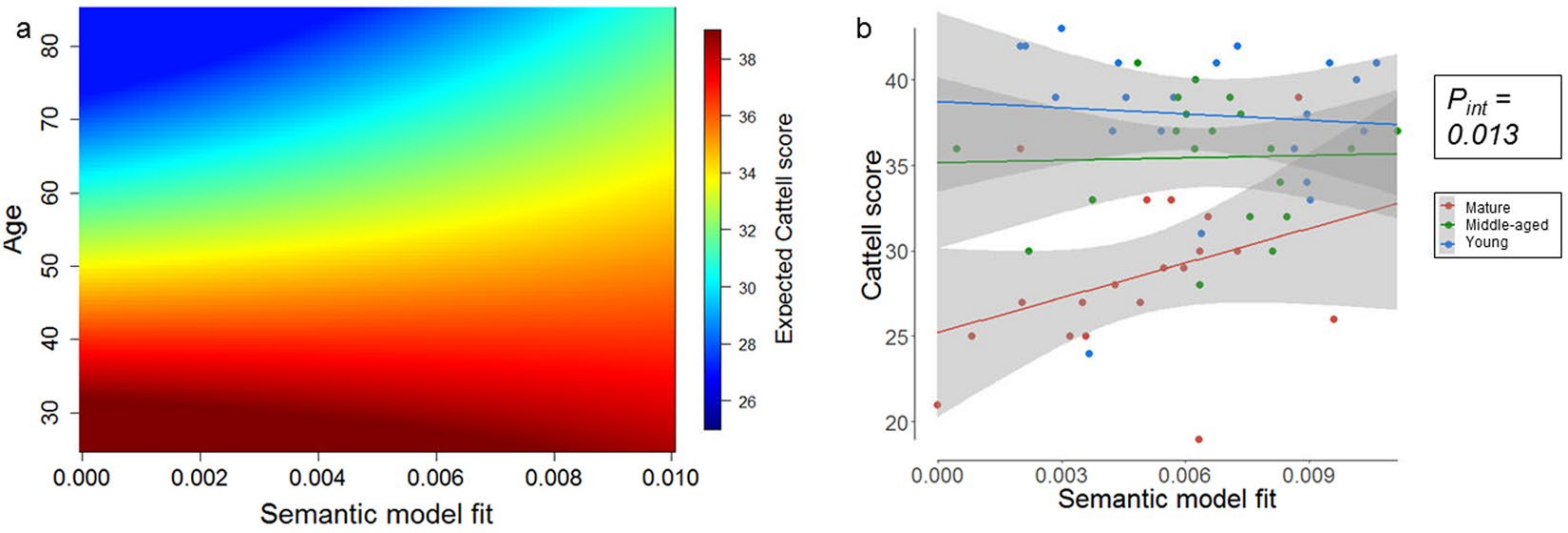
Prediction of fluid intelligence: (ab) interaction between age and the semantic model fit. (**a**) The interaction effect is visualized by generation of the predicted Cattell Score for every combination of age and semantic model fit based on the interaction term from the moderation model. (**b**) The correlation within the young, middle-aged and mature group.

We evaluated whether age moderates the relationship between the visual or semantic model fit and measures of cognition (fluid intelligence, crystallized intelligence, naming accuracy). Fluid intelligence could be predicted from a moderation model including age, the semantic model fit and the interaction of age and the semantic model fit (R² = 0.575, F(80, 4) = 27.0, p < 0.001, Table 1). The main effect of age was significant (β = −0.355, p < 0.001), but the main effect of the semantic model fit was not (β = −898, p = 0.072). Critically, the interaction between age and the semantic model fit was significant (β = 20.5, p = 0.013) (Fig. 6a, Table 1). Visualization of this relationship for a subsample divided into young, middle-aged and mature groups, shows that the relationship between fluid intelligence and semantic model fit becomes stronger for older individuals, i.e. high fluid intelligence in old age is associated with high semantic model fit (Fig. 6b). A trend for significance was found for the interaction between age and visual model fit (β = 2.66, p = 0.062, Table 2), that produced a qualitatively similar effect. No moderation effects were seen in relation to naming accuracy or crystallized intelligence using the semantic model fit (Table 1) or visual model fit (Table 2).

**Table 1:** Moderation analysis: behavioural performance predicted from age, semantic model fit and the interaction of age and the semantic model fit.

	R²	P	Semantic model fit		Age		Interaction	
			β	P	β	P	β	P
Total accuracy	0.302	<0.001	96.7	0.851	−0.152	0.005	5.89	0.486
Fluid intelligence	0.575	<0.001	−898	0.072	−0.355	<0.001	**20.5**	**0.013**
Crystallized intelligence	0.017	0.843	372	0.535	0.052	0.403	−4.23	0.667

Significant results for the interaction effect (P < 0.05) are marked in bold.

**Table 2:** Moderation analysis: behavioural performance predicted from age, visual model fit and the interaction of age and the visual model fit.

	R²	P	Visual model fit		Age		Interaction	
			β	P	β	P	β	P
Total accuracy	0.267	<0.001	−48.5	0.484	−0.207	0.004	1.71	0.247
Fluid intelligence	0.547	<0.001	−106	0.116	−0.374	<0.001	2.66	0.062
Crystallized intelligence	0.022	0.775	−37.9	0.628	−0.024	0.766	1.22	0.464

Significant results for the interaction effect (P < 0.05) are marked in bold.

### Impact of peak visual latency on visual and semantic information

In addition to the amplitude of the visual model fits, the peak latency of the visual model fit was calculated for every subject to test if the speed of visual information processing related to age. Second, we tested if the speed of processing related to the capacity to represent visual and semantic information, as measured by the model fits.

**Figure 7:**
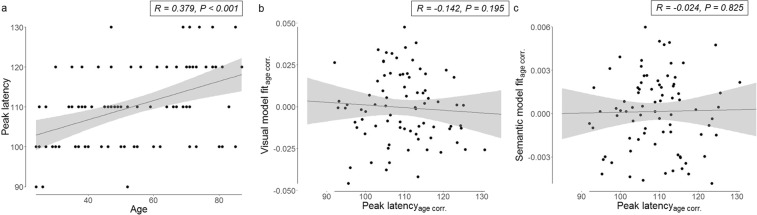
Relationship between the peak latency, age and the visual and semantic model fits. (**a**) Correlation between age and peak latency, (**b**) Correlation between peak latency and visual model fit (corrected for age), (**c**) Correlation between peak latency and semantic model fit (corrected for age).

The average peak of the visual model fit across all participants occurred at 110 ms. The latency of individual participants’ visual model fit peaks increased significantly with age (r = 0.379, p < 0.001; Fig. 7a), showing age-related delays in the visual processing of complex objects. We next tested whether the peak latency of the visual model influences the visual and/or semantic model fits. Since both the peak latency and the visual and semantic model fit are negatively affected by age, the following analysis was corrected for age. Peak latency showed no correlation with the visual model fit (r = −0.142, p = 0. 195) (Fig. 7b) or the semantic model fit (r = −0.024, p = 0.825) (Fig. 7c).

Like above, correlation analyses were conducted to ask if the relationship between the peak latency of the visual model and measures of cognition (fluid intelligence, crystallized intelligence, naming accuracy) were linked, but we found no evidence of this (p > 0.748). Moderation analyses were conducted to test if the relationship between the peak latency of the visual model and measures of cognition varied as a function of age, but no moderation effects were seen (all p’s > 0.1). Therefore, we find no evidence that neural slowing has a dramatic influence on how visual and semantic information is represented.

## Discussion

We investigated differences in object processing across the adult lifespan in a large population-derived sample of cognitively healthy adults using a well-validated model of object processing in the ventral stream^12^. Here, we (1) characterize visual and semantic processes involved in object processing across the adult lifespan, (2) ask if differences in visual processing impact semantics, and (3) evaluate how measures of visual and semantic representations, which we argue reflect the neural responsivity of the visual and semantic processes, relate to cognitive function. We find clear evidence of differences across the adult lifespan in the representation of visual and semantic information: our results show neural slowing and decreases in measures of representation of visual and semantic information with age, while decreased visual effects also relate to decreased semantic effects. In relation to cognition, we see that higher measures of semantic processing are found in subjects with higher naming accuracy, and that higher semantic processing in older age was associated with increased fluid intelligence scores. Together, our results support a view that maintaining high-levels of neural responsivity is associated with both better task-related performance, and more domain general cognitive functions in line with the brain maintenance hypothesis.

Our results demonstrate a relationship between an individual’s semantic processing and both task-specific and domain-general measures of cognition. We find that higher measures of semantic processing were associated with better naming accuracy (Fig. 5d), showing that the semantic model fits are capturing semantic representations that are related to behaviour. It is well established that picture naming errors increase with age (for a review^41^), and this has been previously linked to phonological retrieval errors^41,42^. Our study adds to this by showing that the semantic processing in the first 400 ms, likely prior to phonological processing, may also contribute to naming errors. We also observed a second relationship between semantic model fit and cognition, where model fit became increasingly related to fluid intelligence with increasing age (Fig. 6). Whilst only significant for the semantic model fits, the effects were qualitatively similar and marginally significant for the visual model fits suggesting that neural responsivity overal becames increasingly related to fluid intelligence with increasing age. This illustrates that the capacity to represent visual or semantic information in neural signals, a measure of the neural responsivity of the visual system, could be relevant to a general measure of cognition.

Increased neural responsivity has previously been linked to higher fluid intelligence^31,43^ and cognitive control^44^. Samu *et al*.^31^ reported that mean-task responsive (MTR) components (also a measure of neural responsivity) that related to task performance showed significant age-related declines. The MTR components in Samu’s study gave an aggregate measure of fMRI task responsivity during either picture naming or a fluid intelligence task, and were able to explain individual variability in task performance. These task related activations further declined with age, and increased with task performance. The majority of voxels contributing to the MTR components were from occipitotemporal cortex, with the implication being that the greater task responsivity, the better that performance will be maintained into older adulthood.

Based on our model fits, which we view as measures of neural responsivity derived from MEG data, we find additional evidence that better neural responsivity plays a role in healthy cognitive ageing. This is further supported by correlations we observe between our model fits and MTR components from Samu *et al*.^31^ for the same participants (data for 63/85 of our participants also in^31^). There was a strong correlation between the MTR of the fMRI picture naming task and the visual model fit of the same participants in the MEG picture naming task (r = 0.487, p < 0.001). This suggests that the MTR components at least partially reflect the responsitivity of the neural substrate of visual object processing which we derived in this study. In addition, there was a correlation between the MTR of the fMRI picture naming task and the MEG semantic model fit (r = 0.274, p = 0.030). Overall, this provides additional evidence that the model fits are estimates of neural responsivity. Our analyses are consistent with the idea that better cognitive performance is supported by good neural responsivity. We hypothesize that a reduced ability to modulate task-relevant brain networks may contribute to age-related declines in cognition. Like Samu *et al*.^31^ our results are consistent with the brain maintenance hypothesis, which states that individual differences in age-related brain changes, such as neural responsivity, allow some people to show little or no age-related cognitive decline^32^. Thus, retaining youth-like neural function is key to preservation of cognitive performance across the lifespan^45^.

Another mechanism which is sometimes proposed to compensate for potential age-related changes is the recruitment of contralateral and prefrontal regions^46,47^. Our study does not allow us to differentiate between maintenance and compensation as the mechanism by which some mature controls perform at similar levels to the younger groups. The focus in our study is the timing and untangling of visual and semantic effects, and did not examine regional effects which would be required to test for top-down compensation mechanisms or the recruitment of additional regions. To the contrary, we elected to avoid assumptions about the localization of our effects at the individual level and used data from all available sensors. Our approach leaves open the possibility of a top-down modulatory process on early visual activity, which would be in line with compensation mechanisms. This notion is supported by connectivity studies showing increased frontal to posterior connectivity during object naming in older adults^48,49^. However, our MEG effects did correlate with fMRI-based MTR components that are localized to occipitotemporal cortex which may not be compatible with compensation, suggesting our results are more consistent with the brain maintenance hypothesis than compensation.

Several lines of research suggest an age-related slowing of neural responses to visual stimuli^22,24–28^. Consistent with this, we demonstrate a clear increase in the delay in visual information processing with increasing age, but found no evidence this delay related to age-related cognitive changes. This may argue against the universality of the general slowing hypothesis, which proposes that general slowing leads to age-related declines in performance^50^. Instead, our data argues that although visual slowing does occur across the adult lifespan, it does not necessarily have detrimental consequences for cognition, while the magnitude of the visual and semantic model fits does relate to both task-specific and domain general measures of cognition. However, it has also been noted that age-related processing speed declines may only impact cognition in task with high cognitive demands^51–53^. In the current study, participants are naming a series of highly familiar, easily nameable pictures, and it could be the case that the age-related visual delay we observed would only have cognitive impacts in more challenging situations.

Our finding that the capacity to represent visual and semantic information is lower in mature adults, might be viewed as supporting evidence for the information degradation hypothesis^54^. Repeatedly, correlations have been observed between visual perceptual decline and cognitive decline across the adult lifespan in large samples^55–57^. The information degradation hypothesis states that degraded perceptual input resulting from age-related neurobiological changes causes a decline in cognitive processes^54^. We find that the capacity to represent visual information correlates with the capacity to represent semantic information, which is consistent with this hypothesis. Because our approach is correlational, we cannot make any claims about the causal nature of the changes in neural responsitivity to visual input on semantic processing. To support the information degradation hypothesis and rule out e.g. the influence of cognition on perceptual processing or other confounding effects, experimental manipulation of perceptual input is required^58^. However, our approach does yield a sensitive method to determine neural responsitivity to visual input at the individual level, which can benefit further work aimed at corroborating or refuting the information degradation hypothesis.

Even though we have made use of a large sample of healthy adults from the population-representative Cam-CAN cohort^34^, we acknowledge the need for longitudinal research to further examine the hypothesis that neural responsivity decreases across the lifespan, and that these changes have an impact on cognitive function. From our cross-sectional sample, we can only assess age-related differences^59^. The relationships which we observe do not allow us to make causal inferences and might also underestimate nonlinear age trends^60^. Secondly, our findings offer only a partial explanation for the variability in naming accuracy and fluid intelligence in older adulthood. Note that we investigated visual and semantic processing during picture naming, but not phonological retrieval and articulatory response generation. A future direction which might explain additional variability in naming accuracy consists of the implementation of explicit phonological and articulatory models to elucidate these 2 processes. A consideration is the relatively high education level across individuals in our sample. The limited variability of education levels across the age ranges precludes claims about the effect of education on brain maintenance. Importantly, including education as a covariate of no interest did not change our results, suggesting that the observed findings are beyond the effects of education. Specifically targeted large population-based samples are needed to investigate this in more detail.

In conclusion, our results show that in healthy elderly adults, visual object processing is slower and the capacity of the brain to represent visual and semantic object information is reduced. In elderly participants, having higher measures of neural responsivity were linked to better measures of fluid intelligence, and higher semantic neural responsivity was associated with higher naming accuracy. These results are in line with the brain maintenance hypothesis, which states that individual differences in age-related brain changes allow some people to show little or no age-related cognitive decline. Our measures of neural responsivity suggest that age-related declines may partly be underpinned by a reduced ability to modulate task-relevant brain networks.

## Methods

### Participants

One hundred and eighteen members of the CamCan cohort of healthy adults aged 18–88 years^34^ participated in this study. Exclusion criteria for the Cam-CAN Phase III cohort, that was selected for extensive neuroimaging, included Mini Mental State Examination scores <25^61^, poor vision (<20/50 on the Snellen test^62^), non-native English speakers, drug abuse, a serious psychiatric condition or serious health conditions (for full exclusion criteria, see^34^). Informed consent was obtained from all participants and ethical approval for the study was obtained from the Cambridgeshire 2 (now East of England-Cambridge Central) Research Ethics Committee. All experiments were performed in accordance with relevant guidelines and regulations.

From this subset, 85 participants were included in the current analysis. They were all right-handed and were aged 24–87 years (M = 53.2, SD = 18.0, 44 male). Of the initial total of 118 participants, 19 were excluded because of technical problems during data acquisition, 12 were excluded at the preprocessing stage because of poor data quality (see MEG preprocessing) and 2 were excluded because they were strictly left-handed (assessed by means of the Edinburgh Handedness Inventory). The overall education level in this subset of the population-derived cohort was high: 70.2% obtained a degree, and 88.2% obtained at least an A-level certification. In our sample, age negatively correlated with education level (r: −0.365, p < 0.001). The average score on the HADS depression scale was 2.48 (s.d. 2.89) and on the HADS anxiety scale 4.73 (s.d. 3.34), and these scores did not correlate with age in our dataset (p > 0.167).

### Experimental design

Participants named pictures of single objects at the basic-level (e.g., “tiger”,”broom”). The stimulus set is the same as in Clarke *et al*.^4^ and consisted of 302 items from a variety of superordinate categories that represented concepts from an anglicized version of a large property generation study^18,40^. The items were presented as colour photographs of single objects on a white background. Each trial began with a black fixation cross (500 ms), followed by presentation of the item (500 ms). Afterwards a blank screen was shown, lasting between 2400 and 2700 ms. Each item was presented once. The order of stimuli was pseudo-randomized such that consecutive stimuli were not phonologically related (i.e., shared an initial phoneme) and no more than 4 living or non-living items could occur in a row. Stimuli were presented using Eprime (version 2; Psychology Software Tools, Pittsburgh, PA, USA) and answers were recorded by the experimenter. Offline, responses were checked for accuracy (synonyms, e.g. “couch” for “sofa”, were scored as correct).

Crystallized and fluid intelligence tests were administered offline during a prior stage of the Cam-CAN study^34^. Crystallized intelligence was measured using the Spot the Word test in which participants performed a lexical decision task on word-nonword pairs (e.g. pinnace-strummage)^37^. This test was designed to measure lifetime acquisition of knowledge. Fluid intelligence was measured using the Cattell Culture Fair, Scale 2 Form A, a timed pen-and-paper test in which participants performed 4 subtests with different types of nonverbal puzzles: series completion, classification, matrices and conditions^38^.

### Stimulus measures

Visual information for each item was derived from the AlexNet deep convolutional neural network model^39^, as implemented in the Caffe deep learning framework^63^, and trained on the ILSVRC12 classification data set from ImageNet. We used the layers 2 to 7 of the DNN, consisting of five convolutional layers (conv2–conv5) followed by two fully connected layers (fc6 and fc7). The convolutional kernels learned in each convolutional layer correspond to filters receptive to particular kinds of visual input (conv1 was discarded because conv2 has been shown to mimic the activity in early visual cortex more closely than conv1^8,19^). We presented our 302 stimuli to the DNN which produced activation values for all nodes in each layer of the network for each image. Activation values for all nodes were concatenated across layers, resulting in an objects by nodes matrix. PCA reduction was used to obtain 100 components, otherwise the blank space surrounding objects would be represented across a large number of nodes^64^.

The semantic measures used were the same as those used as in Clarke *et al*.^4^, and derived from semantic feature norms^18,40^. For every concept, these feature norms consist of an extensive list of features generated by participants in response to this concept. These features are visual, auditory, tactile, encyclopedic, etc. The relationship between items can be captured through the similarity of their features, where similar concepts will share many features, while the distinctive properties of a concept will differentiate it from other category members. For each of the 302 concepts, a binary vector indicates whether semantic features (N = 1510) are associated with the concept or not. PCA was used to reduce the concept-feature matrix from 1510 features for every concept, to 6 components for every concept.

### MEG/MRI recording

MEG and MRI acquisition in the Cam-CAN cohort is described in detail in Taylor *et al*.^65^. Continuous MEG data were recorded using a whole-head 306 channel (102 magnetometers, 204 planar gradiometers) Vector-view system (Elekta Neuromag, Helsinki, Finland) located at the MRC Cognition and Brain Sciences Unit, Cambridge, UK. Participants were in a seated position. Eye movements were recorded with electro-oculogram (EOG) electrodes. ECG was recorded by means of one pair of bipolar electrodes. Five head-position indicator (HPI) coils were used to record the head position within the MEG helmet every 200 ms. The participant’s head shape was digitally recorded using >50 measuring points by means of a 3D digitizer (Fastrak Polhemus, Inc., Colchester, VA, USA) along with the position of the EOG electrodes, HPI coils and fiducial points (nasion, left and right periauricular). MEG signals were recorded at a sampling rate of 1000 Hz, with a highpass filter of 0.03 Hz. If required, participants were given MEG-compatible glasses to correct their vision.

### MEG preprocessing

Initial preprocessing of the raw data used MaxFilter version 2.2 (Elekta-Neuromag Oy, Helsinki, Finland) as described in the Cam-CAN pipeline^66^. For each run, temporal signal space separation^67^ was applied to remove noise from external sources and from HPI coils for continuous head-motion correction (correlation threshold: 0.98, 10 s sliding window), and to virtually transform data to a common head position. MaxFilter was also used to remove mains-frequency noise (50 Hz notch filter) and automatically detect and virtually reconstruct noisy channels.

Further preprocessing was performed using SPM12 (Wellcome Institute of Imaging Neuroscience, London, UK). MEG data were low-pass filtered at 200 Hz (fifth order Butterworth filter) and high-pass filtered at 0.1 Hz (fourth order Butterworth filter). Initial epoching from −1s to 1 s was performed before artifact removal by means of Independent Component Analysis (ICA) using RUNICA^68^. Artifactual components were identified using the SASICA toolbox^69^ consisting of components related to blinks, eye movements, rare events, muscle artifacts and saccades. Spatial topographies of the ICs suggested by SASICA were visually inspected prior to their rejection. Finally, IC epochs were averaged and correlated with a “speech template” curve that was modelled as a sigmoidal curve with a slope starting at 200 ms reaching a plateau at 1200 ms. ICs with a correlation of >0.8 were removed. ICA was applied to magnetometers and gradiometers separately. Following ICA, items that were not correctly named or only named after a hesitation period, were excluded from further analysis at the subject level. Finally, MEG data were baseline corrected (time window: −200 to 0 ms) and cropped to the epoch of interest from −200 ms to 600 ms. Temporal signal-to-noise ratio (tSNR) was calculated as the ratio between the mean and standard deviation for the baseline period. Participants with tSNR < 1 were excluded from further processing (N = 12). No significant tSNR differences were observed between age groups (p = 0.183). Data were downsampled to 100 Hz to obtain manageable computing times.

### Visual model fit

Using lasso linear regression, R^2^ values were calculated that captured how well the MEG signals (dependent variable) were modelled by the AlexNet model (independent variables) (Fig. 2a). Lasso regression was used to avoid overfitting. Lasso regression was implemented using glmnet for Matlab^70^ where the regularization parameter lambda was set using 10 fold cross-validation from a set of 100 potential lambda values defined automatically based on the data. Using the optimal lambda value, R² was calculated for each participant at each timepoint and sensor independently. To derive one model fit value per timepoint, R² values were subsequently averaged across all sensors (magnetometers and gradiometers) to construct a time course for every participant. We averaged across all sensors because visual object processing elicits widespread neural responses and the distribution of these responses might vary between individuals and age groups. For this reason, we did not want to make any assumptions by using predefined regions. To correct for individual differences in model fit unrelated to object processing, we subtracted the average R² values before stimulus onset (−200 to 0 ms) from the R² values after stimulus onset (0 to 600 ms) for every participant. To obtain a measure of each individual’s peak model fit latency, a mean template across all participants was constructed, before each individual’s timecourse was virtually shifted in 10 ms steps relative to the mean template to find the maximal correlation to the template^28^. The individual’s peak latency was calculated from the mean peak latency (110 ms) and the shift needed to maximally correlate to the template (Fig. 2c). The individual visual model fit is the visual model fit averaged across sensors at the individual’s peak latency (Fig. 2c).

### Semantic model fit

In a second step, multiple linear regression was performed between the semantic model and the residuals from the visual model fit (Fig. 2b). A time windows of interest between 150 and 400 ms was derived from Clarke *et al*.^4^ (note that the 14 participants from Clarke *et al*.^4^ are not part of the Cam-CAN cohort). An individual’s semantic model fit was calculated by averaging across time points and sensors between 150 and 400 ms. In this way, we are modelling semantic information in a very stringent way, that is over and above what the AlexNet model can explain. By regressing out the visual model, all variability which can be explained by the visual model will be removed from the MEG signals.

### Statistical analysis

To test for age-related changes in visual and semantic processing, the measures of visual and semantic model fit, as well as the measure of peak latency, were correlated with age. Secondly, we investigated the relationships between peak latency, visual model fit and semantic model fit and added age as a covariate of no interest. Next, we correlated peak latency, visual model fit and semantic model fit on one hand with our cognitive measures (naming accuracy, fluid or crystallized intelligence) on the other hand with age as a covariate of no interest.

Using moderation analysis, we test whether the relationship between visual or semantic model fits and our cognitive measures is different across the age groups. As in Samu *et al*.^31^, we used multiple linear regression with an interaction term to test the potential moderation effect of age on the relation between two other variables^71^. More specifically, if we wanted to investigate the relation between X and Y, and Z is the moderator variable “age” to be tested, we ran a multiple linear regression with Y as the dependent variable, and X, Z and the interaction term XZ as predictor variables. A significantly non-zero coefficient of predictor XZ would in turn indicate a moderator effect of Z (“age”) on the relationship between X and Y. In all correlation and moderation analyses, gender was added as a covariate of no interest^31^. Normality was assessed using Q-Q plots and homogeneity of variances was determined by Fligner-Killeen’s Equality of Variances test.

The statistical analyses were performed using all 85 subjects (24–87 years old), with age treated as a continuous variable. However, visualization of e.g. moderation analysis is not always straightforward. Therefore, for visualization purposes we split the dataset in three equal groups of 21 subjects each which were separated by a ten year age gap to highlight changes between age groups. The youngest group consisted of all participants between 24 and 37 years old (12 female, 9 male), the middle-aged group consisted of all participants between 47 and 60 years old (10 female, 11 male), the oldest group consisted of all participants between 70 and 87 years old (10 female, 11 male).

## Data Availability

The data set analysed in this study is part of the Cambridge Centre for Ageing and Neuroscience (Cam-CAN) research project (www.cam-can.com). The entire Cam-CAN dataset will be made publicly available in the future.
